# Supplementary material for: The Mediator complex regulates enhancer-promoter interactions
Source: Nat Struct Mol Biol. 2023 Jul 10;30(7):991–1000. doi: 10.1038/s41594-023-01027-2 (PMC10352134; doi:10.1038/s41594-023-01027-2)
Supplement: Supplementary file 1 — Reporting Summary [file 41594_2023_1027_MOESM1_ESM.pdf]

Corresponding author(s): A. Marieke Oudelaar

Last updated by author(s): Apr 15, 2023

Reporting Summary

Nature Portfolio wishes to improve the reproducibility of the work that we publish. This form provides structure for consistency and transparency in reporting. For further information on Nature Portfolio policies, see our Editorial Policies and the Editorial Policy Checklist.

Statistics

For all statistical analyses, confirm that the following items are present in the figure legend, table legend, main text, or Methods section.

n/a	Confirmed	
☐	☒	The exact sample size (*n*) for each experimental group/condition, given as a discrete number and unit of measurement
☐	☒	A statement on whether measurements were taken from distinct samples or whether the same sample was measured repeatedly
☐	☒	The statistical test(s) used AND whether they are one- or two-sided<br>*Only common tests should be described solely by name; describe more complex techniques in the Methods section.*
☒	☐	A description of all covariates tested
☐	☒	A description of any assumptions or corrections, such as tests of normality and adjustment for multiple comparisons
☐	☒	A full description of the statistical parameters including central tendency (e.g. means) or other basic estimates (e.g. regression coefficient) AND variation (e.g. standard deviation) or associated estimates of uncertainty (e.g. confidence intervals)
☐	☒	For null hypothesis testing, the test statistic (e.g. *F*, *t*, *r*) with confidence intervals, effect sizes, degrees of freedom and *P* value noted<br>*Give P values as exact values whenever suitable.*
☒	☐	For Bayesian analysis, information on the choice of priors and Markov chain Monte Carlo settings
☒	☐	For hierarchical and complex designs, identification of the appropriate level for tests and full reporting of outcomes
☒	☐	Estimates of effect sizes (e.g. Cohen's *d*, Pearson's *r*), indicating how they were calculated

Our web collection on statistics for biologists contains articles on many of the points above.

Software and code

Policy information about availability of computer code

Data collection	Illumina NextSeq 550.
Data analysis	CapCruncher pipeline v.1 (https://github.com/sims-lab/CapCruncher); MCC pipeline v.1 (https://github.com/jojdavies/Micro-Capture-C), based on scripts available for academic use through the Oxford University Innovation software store (https://process.innovation.ox.ac.uk/software/p/16529a/micro- capture-c-academic/1); Bowtie2 v.2.3.5; HiC-Pro v.2.11.1; oligo design tool v.0.1.1b (https://oligo.readthedocs.io/en/latest/); Samtools v.1.9; MACS2 v.2.1.2; deepTools v.3.0.1; DiffBind v.3.6.5; DESeq2 v.1.36.0; NGseqBasic pipeline v.1 (https://github.com/Hughes-Genome-Group/NGseqBasic/releases).

For manuscripts utilizing custom algorithms or software that are central to the research but not yet described in published literature, software must be made available to editors and reviewers. We strongly encourage code deposition in a community repository (e.g. GitHub). See the Nature Portfolio guidelines for submitting code & software for further information.

Data

Policy information about availability of data

All manuscripts must include a data availability statement. This statement should provide the following information, where applicable:

- Accession codes, unique identifiers, or web links for publicly available datasets
- A description of any restrictions on data availability
- For clinical datasets or third party data, please ensure that the statement adheres to our policy

All raw and processed sequencing data generated in this study are available from the Gene Expression Omnibus (GEO) as a SuperSeries under accession number GSE205984. DNase-I hypersensitivity data and ChIP-Seq data for CTCF are available from ENCODE under accession codes ENCSR000ENM and ENCSR000BSE, respectively. ChIP-seq data for MED26 are available from GEO under accession code GSE121355. TT-seq data are available from GEO under access code GSE139468.

Human research participants

Policy information about studies involving human research participants and Sex and Gender in Research.

Reporting on sex and gender	Not applicable.
Population characteristics	Not applicable.
Recruitment	Not applicable.
Ethics oversight	Not applicable.

Note that full information on the approval of the study protocol must also be provided in the manuscript.

Field-specific reporting

Please select the one below that is the best fit for your research. If you are not sure, read the appropriate sections before making your selection.

☒ Life sciences ☐ Behavioural & social sciences ☐ Ecological, evolutionary & environmental sciences

For a reference copy of the document with all sections, see nature.com/documents/nr-reporting-summary-flat.pdf

Life sciences study design

All studies must disclose on these points even when the disclosure is negative.

Sample size	The data presented in the manuscript represent the averages of three biological replicates. These sample sizes were chosen to generate data at sufficient depth and assess differences between conditions robustly. These sample sizes are sufficient, since the observed biological effects of interest are clearly detectable between conditions and robust across replicates. For Micro-Capture-C experiments, multiple technical replicates for each biological replicate were included to boost the complexity of the data.
Data exclusions	No data were excluded.
Replication	All experiments based on sequencing data were performed for n=3 biologically independent samples as described and all attempts were successful. Immunoblots were performed independently 3 times with similar results.
Randomization	Samples were randomly allocated into different experimental groups prior to their treatment with dTAG ligand or DMSO.
Blinding	All samples were analyzed with the same pipelines, in which results are generated by scripts without interference of the researchers. Since potential expectations of the researchers cannot influence the data analysis and results, blinding is not relevant to this study.

Reporting for specific materials, systems and methods

We require information from authors about some types of materials, experimental systems and methods used in many studies. Here, indicate whether each material, system or method listed is relevant to your study. If you are not sure if a list item applies to your research, read the appropriate section before selecting a response.

Materials & experimental systems

n/a	Involved in the study
☐	☒ Antibodies
☐	☒ Eukaryotic cell lines
☒	☐ Palaeontology and archaeology
☒	☐ Animals and other organisms
☒	☐ Clinical data
☒	☐ Dual use research of concern

Methods

n/a	Involved in the study
☐	☒ ChIP-seq
☒	☐ Flow cytometry
☒	☐ MRI-based neuroimaging

Antibodies

Antibodies used

Rabbit anti-HA-Tag (C29F4) antibody (1:1000 (Immunoblotting), 1 ug (ChIP-seq), Cell Signaling Technology, 3724), Mouse anti-GAPDH (6C5) antibody (1:2000, Abcam, ab8245), Rabbit HRP anti-Histone H3 antibody (1:5000, Abcam, ab21054), Goat anti-Rabbit IgG H&L antibody (HRP) (1:3000, Abcam, ab205718), Goat anti-Mouse IgG H&L antibody (HRP) (1:3000, Abcam, ab205719), Drosophila spike-in antibody (1 ug, Active Motif, 61686), Rabbit anti-SMC1A antibody (1:50, Abcam, ab9262), Guinea pig anti-rabbit secondary antibody (1:100, Active Motif, 53160), Rabbit IgG isotype control antibody (1:50, Cell Signaling Technology, 2729S).

Validation

Validation was performed by the manufacturer. The antibodies were purified using immunogen affinity and validated by immunoprecipitation, immunohistochemical analysis, and western blotting.

Eukaryotic cell lines

Policy information about cell lines and Sex and Gender in Research

Cell line source(s)

Wild type and MED14-dTAG human colorectal carcinoma HCT-116 cells were a gift from Georg Winter (CeMM, Vienna). The MED14-dTAG HCT-116 cells were generated in Jaeger et al, Nature Genetics 2020. Wild type HCT-116 cells were originally obtained from ATCC (CCL-247).

Authentication

The cells were authenticated using the KaryoStat+ assay (Thermo Fisher).

Mycoplasma contamination

All cell lines tested negative for mycoplasma contamination.

Commonly misidentified lines
(See ICLAC register)

No commonly misidentified lines were used.

ChIP-seq

Data deposition

☒ Confirm that both raw and final processed data have been deposited in a public database such as GEO.

☒ Confirm that you have deposited or provided access to graph files (e.g. BED files) for the called peaks.

Data access links
May remain private before publication.

Cut&Tag (GSE205905) and ChIP-seq (GSE225294) data from this study are available from the Gene Expression Omnibus (GEO) under GSE205984 SuperSeries.

Files in database submission

GSM6235280 Cut&Tag DMSO_1
GSM6235281 Cut&Tag DMSO_2
GSM6235282 Cut&Tag DMSO_3
GSM6235283 Cut&Tag DMSO_4
GSM6235284 Cut&Tag DMSO_5
GSM6235285 Cut&Tag dTAG_1
GSM6235286 Cut&Tag dTAG_2
GSM6235287 Cut&Tag dTAG_3
GSM6235288 Cut&Tag dTAG_4
GSM6235289 Cut&Tag dTAG_5
GSM6235290 Cut&Tag IgG_DMSO
GSM6235291 Cut&Tag IgG_dTAG
GSM7043684 ChIP-seq DMSO_1
GSM7043685 ChIP-seq DMSO_2
GSM7043686 ChIP-seq DMSO_3
GSM7043687 ChIP-seq dTAG_1
GSM7043688 ChIP-seq dTAG_2
GSM7043689 ChIP-seq dTAG_3
GSM7043690 ChIP-seq Input_DMSO_1
GSM7043691 ChIP-seq Input_DMSO_2
GSM7043692 ChIP-seq Input_DMSO_3
GSM7043693 ChIP-seq Input_dTAG_1

GSM7043694 ChIP-seq Input_dTAG_2
GSM7043695 ChIP-seq Input_dTAG_3

Genome browser session
(e.g. UCSC)

SMC1A Cut&Tag:
https://genome-euro.ucsc.edu/cgi-bin/hgTracks?db=hg38&lastVirtModeType=default&lastVirtModeExtraState=&virtModeType=default&virtMode=0&nonVirtPosition=&position=chr8%3A127403541%2D128075640&hgsid=289897223_siSABI5BvordAAzIcKzbmk8ElwPq

MED-HA ChIP-seq:
https://genome-euro.ucsc.edu/cgi-bin/hgTracks?db=hg38&lastVirtModeType=default&lastVirtModeExtraState=&virtModeType=default&virtMode=0&nonVirtPosition=&position=chr9%3A21390261%2D21970274&hgsid=296213738_k9i9nlThJHaEvlcOQfaDaJK6pP2T

Methodology

Replicates

Cleavage under targets and tagmentation (CUT&Tag4) experiments were performed for n=3 biologically independent samples (for a total of 5 technical replicates) per experimental condition. ChIP-seq experiments were performed for n=3 biologically independent samples per experimental condition.

Sequencing depth

The samples were sequenced using the NextSeq550 Illumina platform (75-bp paired-end reads) to a sequencing depth of ~10 M reads per sample.

Antibodies

Rabbit anti-SMC1A antibody (1:50 , Abcam, ab9262); Guinea pig anti-rabbit secondary antibody (1:100, Active Motif, 53160); Rabbit anti-HA-Tag (C29F4) antibody (1 ug, Cell Signaling Technology, 3724); Drosophila spike-in antibody (1 ug, Active Motif, 61686).

Peak calling parameters

Paired-end reads were processed for adapter removal and duplicate filtering and mapped to the hg38 reference genome using Bowtie2. Peak calling was performed with MACS2 (consensus peaks with parameter q = 0.1 were selected). All peak profiles were generated using Deeptools.

Data quality

The quality of the data was assessed by comparing the DMSO-treated samples to available SMC1A ChIP-seq data (Rao et al, Cell 2017) and MED26 ChIP-seq data (El Khattabi et al, Cell 2019) in HCT-116 cells.

Software

Paired-end reads were processed for adapter removal and duplicate filtering and mapped to the hg38 reference genome using Bowtie2. Peak calling was performed with MACS2. All peak profiles were generated using Deeptools.
