# Supplementary figures and images for: The Mediator complex regulates enhancer-promoter interactions
Source: Nat Struct Mol Biol. 2023 Jul 10;30(7):991–1000. doi: 10.1038/s41594-023-01027-2 (PMC10352134; doi:10.1038/s41594-023-01027-2)

Extended Data Fig. 1a

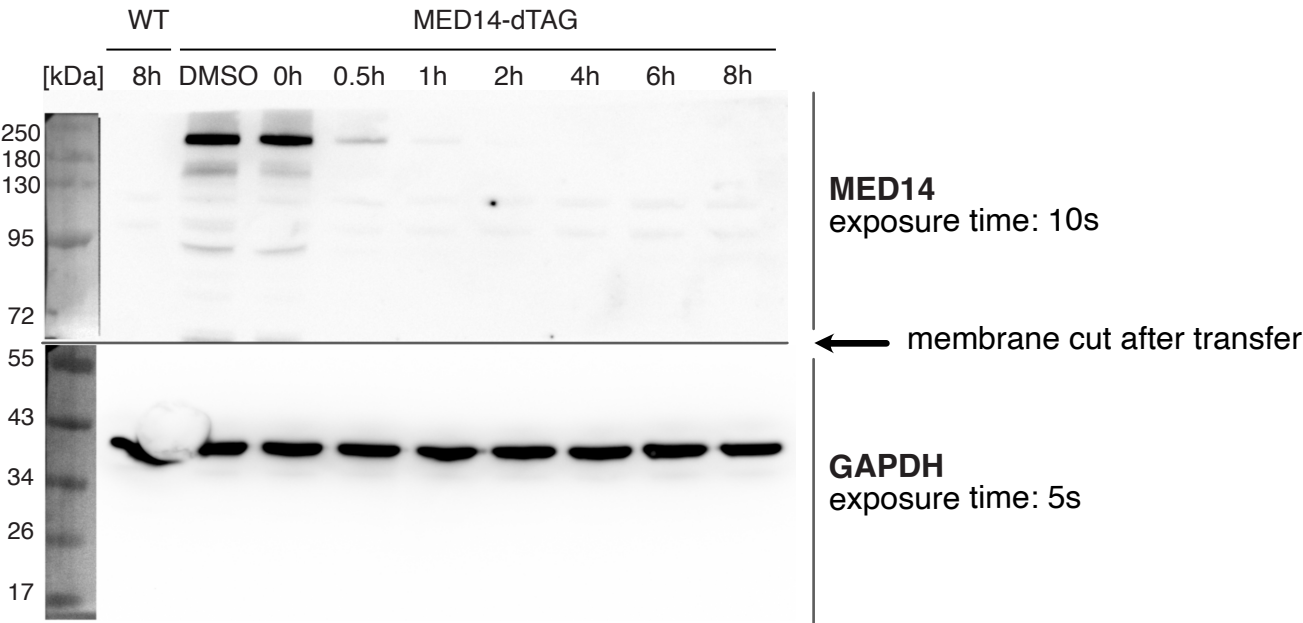

Extended Data Fig. 1b

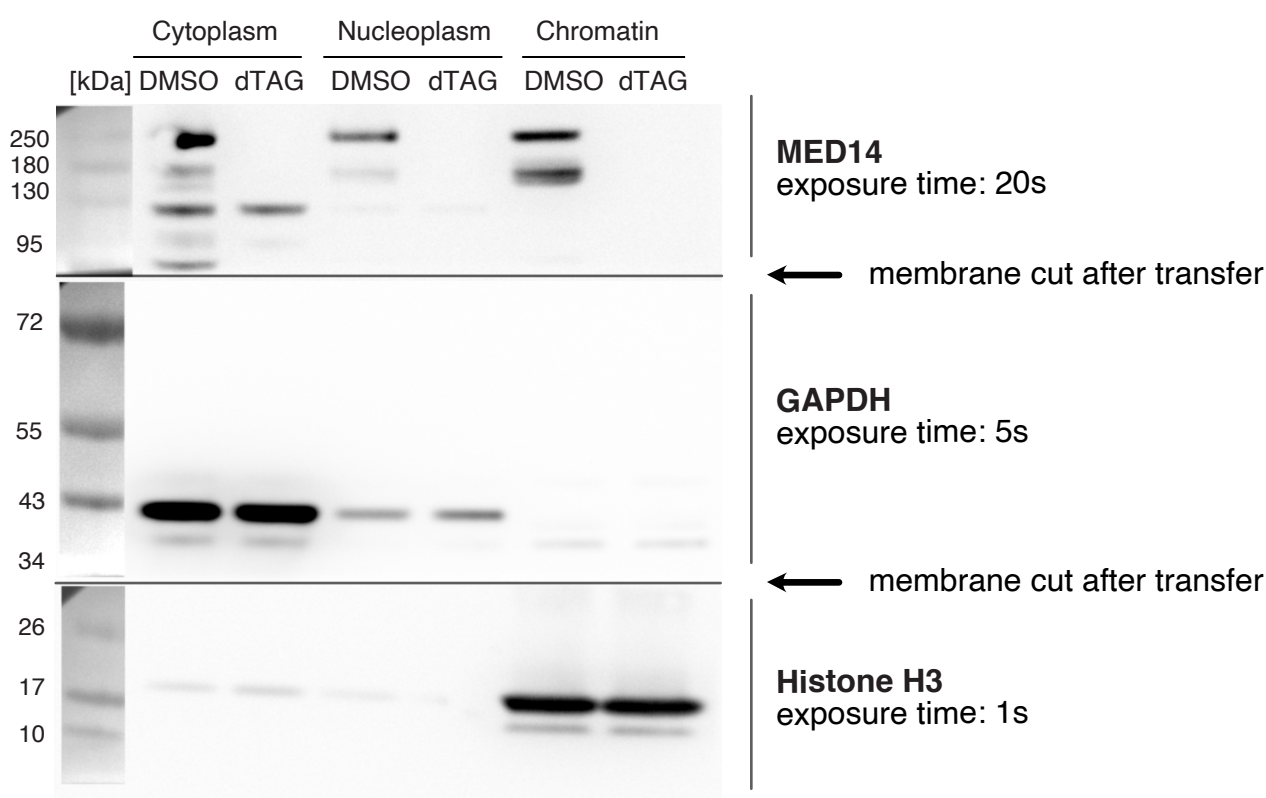

Supplement: Source Data Extended Data Fig. 1 — Unprocessed western blots and/or gels. [file 41594_2023_1027_MOESM5_ESM.pdf]
